# Electronic and Optical Behaviors of Platinum (Pt) Nanoparticles and Correlations with Gamma Radiation Dose and Precursor Concentration

**DOI:** 10.3390/nano16010063

**Published:** 2026-01-01

**Authors:** Elham Gharibshahi, Elias Saion, Ahmadreza Ashraf, Leila Gharibshahi, Sina Ashraf

**Affiliations:** 1 Department of Electrical and Computer Engineering, University of Texas at San Antonio (UTSA), One UTSA Circle, San Antonio, TX 78249, USA; 2 Department of Physics, Faculty of Science, University of Putra Malaysia (UPM), UPM Serdang 43400, Selangor, Malaysia; 3 University of the Incarnate Word School of Osteopathic Medicine (UIWSOM), 7615 Kennedy Hill Dr., San Antonio, TX 78235, USA

**Keywords:** platinum nanoparticles, gamma radiolysis, optical properties, conduction band, precursor concentration, nanostructure

## Abstract

The purpose of this research is to examine how the electro-optical behavior of platinum (Pt) nanoparticles prepared via the gamma radiolysis process is related to both the radiation dose and to the Pt precursor concentration. The Pt precursor used in these experiments has been radiolytically degraded using a ^60^Co gamma source at dosages ranging from 80 kGy to 120 kGy. As well, varying the concentration of the Pt precursor from 5.0 × 10^−4^ M to 20.0 × 10^−4^ M was carried out as a systematic investigation. Spectrophotometric analysis utilizing UV–Visible spectroscopy and TEM provided the optical data and particle size information for the nanoparticles. The results indicate that increasing the radiation dosage results in smaller Pt nanoparticle sizes due to an increased rate of nucleation and that increasing the Pt precursor concentration leads to larger Pt nanoparticles due to an increase in ion recombination. Both the dose and concentration dependency of the optical absorption spectrum indicate a significant relationship between size and plasmon behavior. Also, the conduction band energy level, which was determined from the maximum of the UV–Visible absorption peak, is dependent on the particle size and shows a pronounced quantum confinement effect, with the conduction band energy increasing as the particle size decreases. Thus, these studies provide a definitive correlation of structure–property in Pt nanoparticles and confirm the capability of the gamma radiolytic synthesis process to be used for controlling the specific electronic and optical properties of Pt nanoparticles.

## 1. Introduction

There is considerable interest in platinum nanoparticles (Pt NPs) because they have unique electrical and optical characteristics that are very different from those of bulk platinum, so they are applicable in many areas such as catalysis, sensing technology, energy conversion, and medical technology [1,2,3,4]. The reason why Pt NPs have an excellent catalytic activity in the area of fuel cells, hydrogenation reactions, and automotive exhaust systems is that Pt NPs have a high surface-to-volume ratio and their surface electronic structure can be controlled [5,6]. In addition to their catalytic activity, Pt nanoparticles are incorporated into glucose and gas sensors and studied in relation to therapeutic and diagnostic applications because of their chemical stability and biocompatibility [7,8,9,10,11].

The choice of synthesis method has a substantial effect on the physical, electrical, and optical properties of Pt nanoparticles. There are a variety of synthesis routes available for producing Pt nanoparticles, including chemical reduction, sol–gel, polyol, microemulsion, and electrochemical deposition. These routes include the gamma radiolytic route, which is advantageous compared to the other routes. Using the gamma radiolytic route, it is possible to produce pure Pt nanoparticles without using chemical reducing agents and to control precisely the particle size and the distribution of particles [12,13,14,15,16,17]. During radiolytic synthesis, by altering the dose of gamma radiation applied and the precursor concentration, it is potential to associate these parameters directly to the variations in electronic structure and optical reaction of Pt nanoparticles [18,19]. Studies conducted previously have demonstrated that an increase in the dose of gamma radiation used results in a decrease in particle size and an increase in optical absorption and that a change in precursor concentration affects the number density and aggregation kinetics of particles [20].

The production of nanoparticles and nanostructures using a high-energy source has recently been generalized as High-Intensity Power (HIP) processing, where a large energy density (energy per unit volume) is utilized in a short space of time, i.e., high power. This creates a non-equilibrium processing field and covers microwave irradiation to X-rays and gamma radiation with energy levels in the MeV range. In HIP processing, depending on the detail of the process, these particles can aggregate and form nanostructured materials, as indeed cited by the authors [8]. Such structures have been observed previously in quantum catalysts processed via the HIP method [21].

The optical behavior of metal nanoparticles has been of interest for some time due to their localized surface plasmon resonance (LSPR) and characteristic extinction spectra [22,23]. However, the situation regarding Pt nanoparticles is particularly interesting. Compared to Au and Ag nanoparticles, whose optical absorption spectra are well characterized, Pt nanoparticles demonstrate less defined optical absorption spectra, with absorption peaks declared in the UV and visible spectral ranges [24].

This ambiguity in absorption features is indicative of size- and shape-dependent effects, as well as the influence of electronic band structure and quantum confinement, e.g., when the particle size is decreased to the point where discrete electronic states appear, the absorption spectrum shifts and electronic transitions are modified [2,25,26]. These factors demonstrate the need for studying the relationships between gamma dose, precursor concentration, and the electronic and optical behaviors of Pt nanoparticles and for providing insight into their structure–property relationships at the nanoscale.

This paper presents a comprehensive study of the electronic and optical behaviors of Pt nanoparticles synthesized via gamma radiolysis. The focus of this study is to identify the correlations between these behaviors and two key synthesis parameters: radiation dose and precursor concentration. Through the combination of experimental characterization techniques and theoretical interpretations, our objective is to develop new knowledge regarding the electronic transitions that govern the optical responses of Pt nanoparticles and to develop structure–property relationships that will contribute to both fundamental knowledge and practical uses of these nanomaterials [27,28,29]. The novelty of this work lies in establishing direct correlations between particle size, radiation dose, precursor concentration, and the resulting conduction band energies of Pt nanoparticles, which has not been comprehensively examined in previous radiolytic studies. This study also introduces a dose-dependent sensitivity parameter that quantitatively describes how precursor concentration affects particle size, providing a new analytical approach to understanding radiolytic growth mechanisms. Furthermore, the combined use of conduction band analysis, UV–Vis spectra, and TEM confirms previously unreported quantum confinement behavior in Pt nanoparticles synthesized under varying radiolytic conditions. This research looks at how the dose of radiation and the concentration of precursors affect the size of platinum nanoparticles, their electronic properties, and how they behave optically. The goal is to find connections that explain the relationship between the structure and properties of these nanoparticles at the nanoscale.

## 2. Materials and Methods

### 2.1. Chemicals

Sigma Aldrich (St. Louis, MO, USA) provided all chemicals as received. Tetraammine Platinum Chloride Hydrate [(Pt (NH_3_)_4_]Cl_2_·H_2_O) was utilized as the platinum source; polyvinyl pyrrolidone (MW ≈ 29,000) as the stabilizing agent; isopropyl alcohol (IPA) as a scavenger for radical species; and tetrahydrofuran (THF) and deionized water as solvents.

### 2.2. Nanoparticle Preparation

Selected masses of [Pt(NH_3_)_4_]Cl_2_·H_2_O were dissolved in 50 mL of THF. In a separate beaker, 2 wt% PVP was dissolved in 150 mL of deionized water. The solutions containing the precursor and polymer were then combined, followed by the addition of 20 mL of IPA. The mixture was permitted to stir for 1 h and degassed under a flow of nitrogen gas (99.5% purity) for 1 h to eliminate dissolved oxygen. The precursor solution was transferred into sealed vials containing approximately 33 mL of solution each. The vials were then irradiated with gamma radiation (^60^Co) provided by a J.L. Shepherd source at the Malaysian Nuclear Agency. Irradiation was performed at a dose rate of 2.9 kGy/h, with irradiation times ranging from approximately 28 to 41 h depending on the total absorbed dose.

Gamma ray energies of 1.17 MeV and 1.33 MeV (average = 1.25 MeV) are produced by this source. Absorbed doses of 80 kGy, 90 kGy, 100 kGy, 110 kGy, and 120 kGy were achieved. Dosimetry measurements were made with the use of the Fricke system, and corrections for radioactive decay were also applied.

### 2.3. Characterization

Optical absorption spectra of the irradiated samples were measured with a UV–Vis spectrometer (Shimadzu UV-1650PC, Shimadzu Corporation, Kyoto, Japan). Transmission electron microscopy (TEM) images of the particle size distributions were obtained using a transmission electron microscope (HITACHI H-7500, Hitachi, Ltd., Tokyo, Japan) operating at 100 kV. A droplet of the irradiated colloidal dispersion was placed on a carbon-coated copper grid, dried at room temperature, and analyzed by TEM.

## 3. Results and Discussion

### 3.1. Effect of Initial Ion Concentration on Absorbance and Particle Size

**Figure 1:**
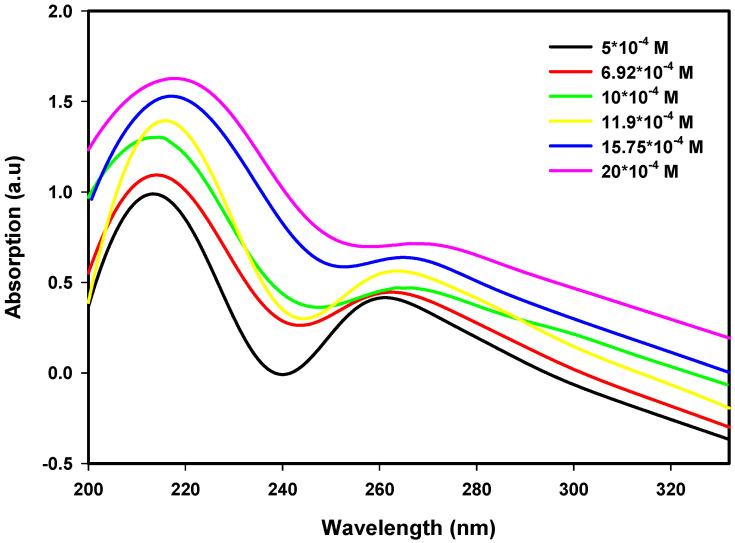
UV–Visible spectra of Pt nanoparticles at 80 kGy for various ion concentrations.

**Figure 2:**
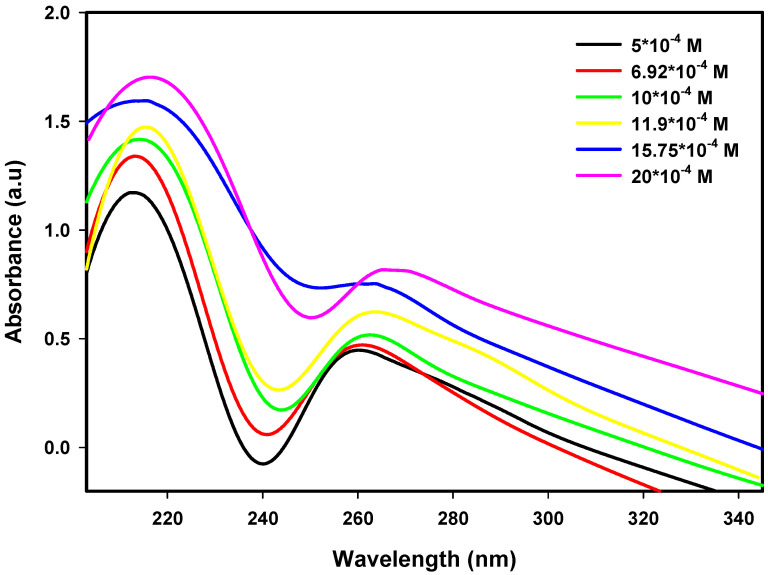
UV–Visible spectra of Pt nanoparticles at 90 kGy for various ion concentrations.

**Figure 3:**
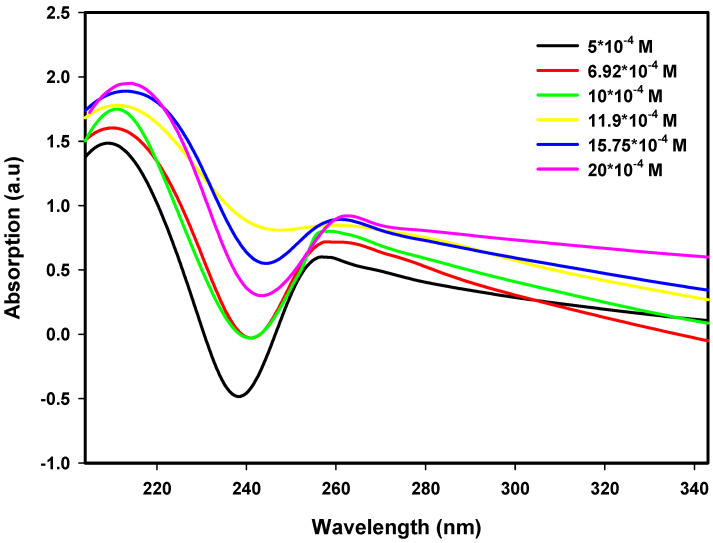
UV–Visible spectra of Pt nanoparticles at 120 kGy for various ion concentrations.

Pt nanoparticle UV–Vis absorption spectra were obtained over an ion concentration range of 5.0 × 10^−4^ M to 20.0 × 10^−4^ M and radiation dose range of 80 kGy to 120 kGy. An increase in ion concentration causes an increase in absorbance, indicating that a larger number of Pt nanoparticles form with the reduction of Pt^2+^ ions, whereas the absorption maxima are shifted towards longer wavelengths, indicating particle growth. The trends illustrated in Figure 1, Figure 2 and Figure 3 indicate that a slight increase in dose also leads to an increase in absorbance intensity as well as a blue shift of the absorption maxima. As such, the results provide evidence on how both the precursor concentration and radiation dose influence nanoparticle nucleation and growth, as well as their optical properties, to provide insight into the structure–property relationships of Pt nanoparticles.

**Figure 4:**
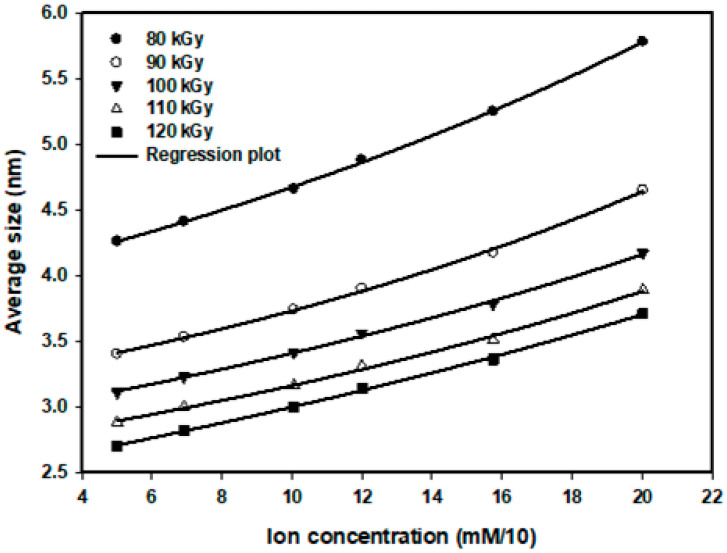
Relationship between precursor ion concentration and average particle size of Pt nanoparticles synthesized at various radiation doses.

An exponential increase in the mean particle size as a function of increased ion concentration was demonstrated in Figure 4 and may be described using the following empirical relationship:

d=dmin+B expCC0

where d is the mean particle diameter formed at ion concentration C, $d_{min}$ is the mean diameter formed at the lowest ion concentration $5.0\times 10^{-4}$ M, B is a constant, and $C_{0}$ is a dose sensitivity parameter indicating how the particle size of Pt nanoparticles forms based on ion concentration. The value of $C_{0}$ is determined from the slope of $\ln\left(d-d_{min}\right)$ vs. C. Sensitivity values were calculated for each radiation dose as $26.596\times 10^{-4}\;M$, $21.882\times 10^{-4}\;M$, $28.986\times 10^{-4}\;M$, $26.525\times 10^{-4}\;M$, and $39.526\times 10^{-4}\;M$ for doses of 80, 90, 100, 110, and 120 kGy, respectively.

The value of $C_{0}$ is influenced mainly by two factors: the nucleation process and the number of unreduced ions. At lower ion concentrations, the nucleation rate is lower than the availability of initial ions, resulting in smaller particle sizes. With an increasing ion concentration, the probability of ion recombination becomes higher, leading to the formation of larger particles, as illustrated in the figure.

### 3.2. Influence of Dose on Conduction Band Energy

Pt nanoparticles’ conduction band energy was computed from the highest wavelength (λ) of UV–Visible absorbance peaks using the equation $E=\frac{hc}{\lambda_{max}}$, where h is Planck’s constant, and c is the speed of light [15,30,31,32]. The rise in the conduction band energy is a result of a decreasing particle size as the radiation dose increased.

**Figure 5:**
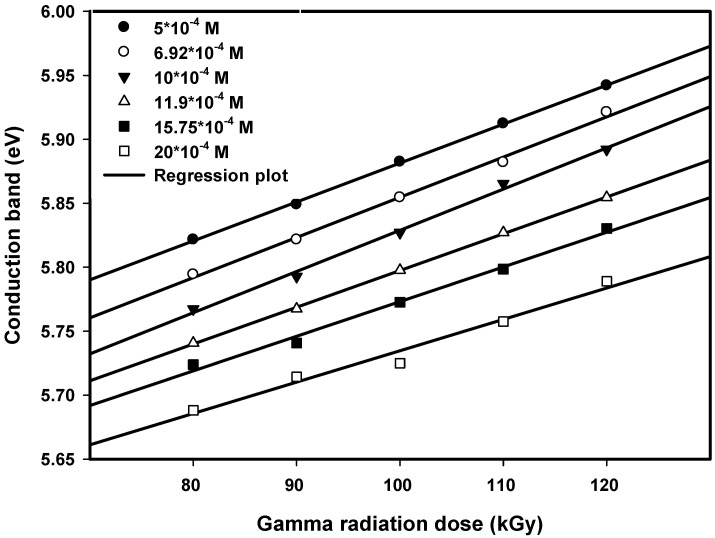
Conduction band energy of Pt nanoparticles for the first peak of different concentrations versus dose. The linear lines are drawn to fit the experimental data.

**Figure 6:**
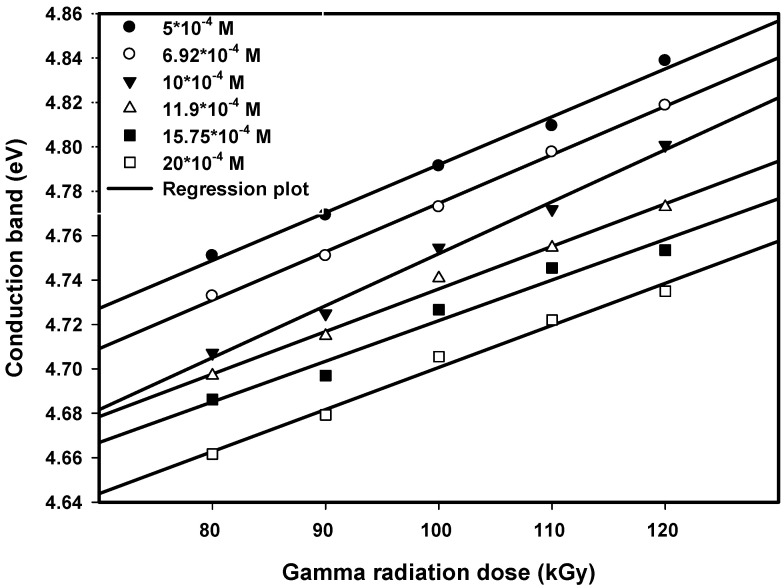
Conduction band energy of Pt nanoparticles for the second peak of different concentrations versus dose. The linear lines are drawn to fit the experimental data.

The data in Figure 5 and Figure 6 illustrate how the conduction band energy changed with radiation dose for both the first and second maxima at various concentrations of initial ions. As the radiation dose increased from 80 to 120 kGy, the first maximum’s conduction band energy rose from an approximate 5.82 to 5.94 eV, while the second maximum’s conduction band energy rose from approximately 4.75 to 4.84 eV over the same dose range.

The more pronounced behavior in conduction band energy between 80 and 90 kGy can be attributed to a critical threshold in the nucleation and growth processes of Pt nanoparticles. At 80 kGy, the nucleation rate is relatively low, allowing for larger particle formation and some degree of agglomeration. As the dose increases to 90 kGy, the production of radiolytic reducing species is sufficient to significantly enhance nucleation, leading to a sudden decrease in particle size and corresponding increase in conduction band energy.

Quantum confinement effects were illustrated by these findings, as they clearly demonstrate that the reduced dimensions of smaller particles are associated with larger bandgaps as the electronic structures of the particles are modified by their size. These changes in conduction bands confirm that the radiation dose affects the size-dependent modification of the conduction band of particles.

### 3.3. Influence of Particle Size on Conduction Band Energy

As the energy of the conduction band increases, this directly influences the decrease in particle size as the dose increases and the concentration of ions decreases. The mechanism of aggregation of particles is governed by two competing processes: nucleation and ion recombination. Nucleation rates will exceed recombination rates when the dose is increased to high levels, thus yielding smaller particles. On the other hand, when the dose is decreased to low levels, the recombination process will dominate the nucleation process, thus creating larger particles. Likewise, at a constant dose level, the concentration of ions will also control whether the recombination process or the nucleation process will be favored. A higher concentration of ions will favor the recombination process and create larger particles; a lower concentration of ions will favor the nucleation process and create smaller particles.

**Figure 7:**
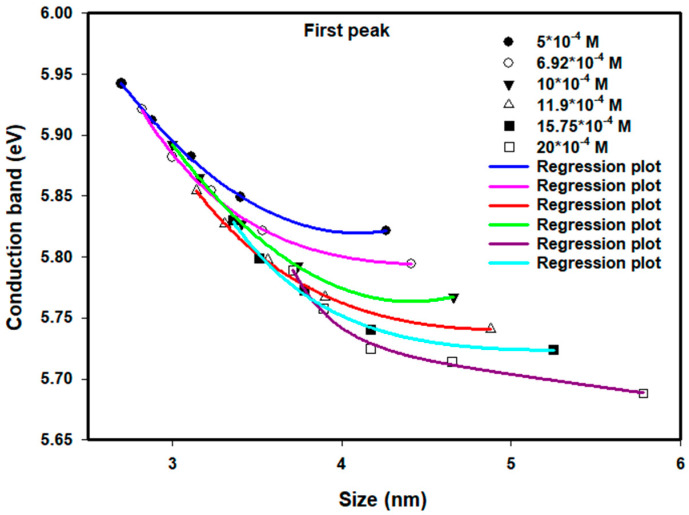
Conduction band of colloidal Pt nanoparticles of different concentrations versus average size for the first peak. The lines of empirical equation are drawn to fit the experimental data.

**Figure 8:**
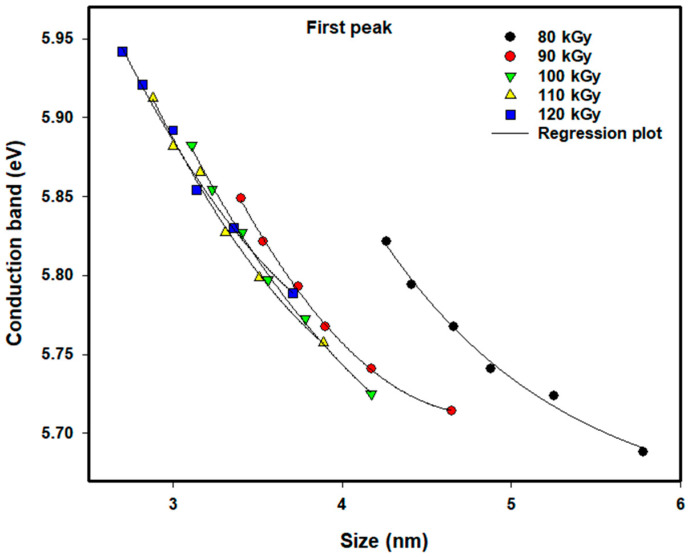
Conduction band of colloidal Pt nanoparticles of different doses versus average size for the first peak. The lines of empirical equation are drawn to fit the experimental data.

Figure 7 and Figure 8 provide the values of the conduction bands for the first absorption peak, where the precursor concentrations ranged between $5.0\times 10^{-4}\;M$ and $20.0\times 10^{-4}\;M$, and the doses ranged between 80 and 120 kGy. Below a particle diameter of ~4.6 nm, the conduction band shows the expected size dependence that metallic nanoparticles should have. As the dose increases (and, therefore, the synthesized Pt nanoparticle size is reduced), the synthesized Pt nanoparticles will exhibit higher conduction band energies. However, the lowest dose (80 kGy) produced particle diameters greater than ~4.6 nm, and consequently, the conduction band exhibited different trends than would be expected. It is thought that insufficient stabilization of the Pt nanoparticles by the PVP used resulted in some degree of agglomeration and/or growth of the Pt nanoparticles, leading to particle diameters outside of what was expected. Further research is necessary to determine the extent of the nucleation and agglomeration processes under these conditions.

**Figure 9:**
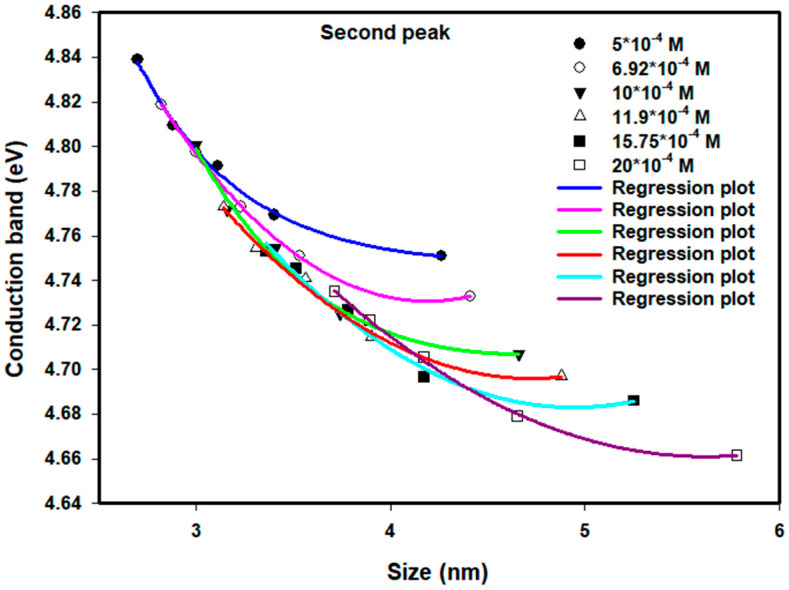
Conduction band of colloidal Pt nanoparticles of different concentrations versus average size for the second peak. The lines of empirical equation are drawn to fit the experimental data.

**Figure 10:**
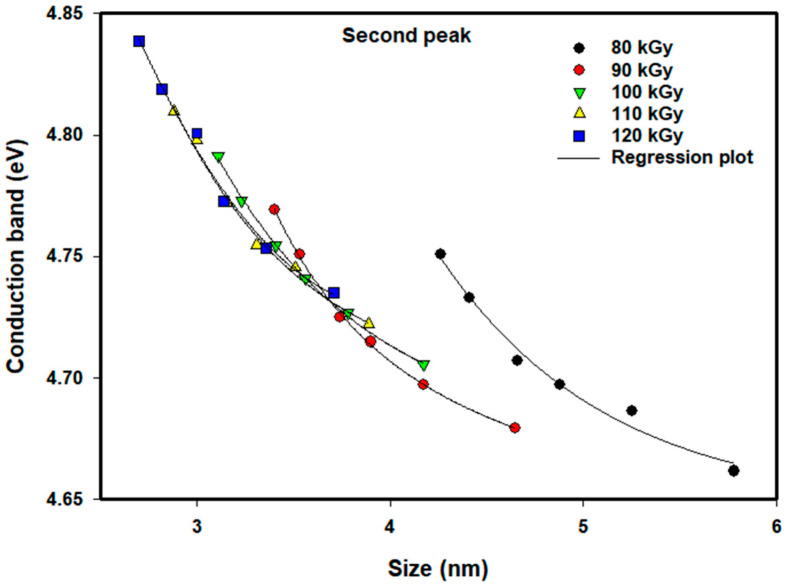
Conduction band of colloidal Pt nanoparticles of different doses versus average size for the second peak. The lines of empirical equation are drawn to fit the experimental data.

Figure 9 and Figure 10 demonstrate the same trend of the values of the conduction bands for the second absorption peak for the same conditions as previously mentioned. Higher doses result in smaller particles that exhibit higher conduction band energies, whereas at the 80 kGy dose, the larger particle diameters (>4.6 nm) again produced results that were less than expected; it is thought that the stabilization of the Pt nanoparticles by the PVP was compromised to some extent, allowing for further agglomeration of the Pt nanoparticles.

### 3.4. Transmission Electron Microscopy (TEM) Analysis

Transmission electron microscopy (TEM) images were used to analyze the size distribution of platinum nanoparticles synthesized with a radiation dose between 80 kGy and 120 kGy and a precursor concentration from $5.0\times 10^{-4}\;M$ to $20.0\times 10^{-4}\;M$.

**Figure 11:**
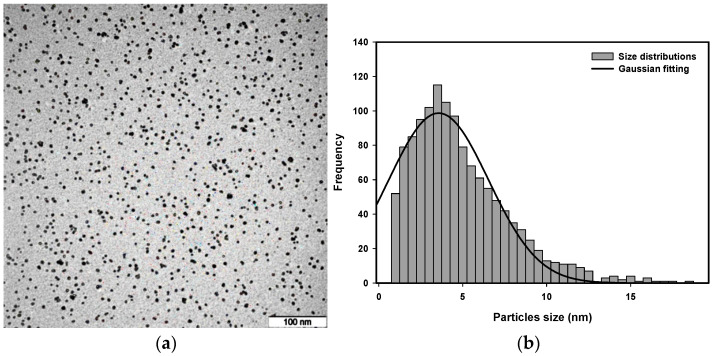
(**a**) TEM micrograph and (**b**) size distribution with Gaussian fitting of Pt nanoparticles of 11.9 × 10^−4^ M irradiated at 100 kGy.

**Figure 12:**
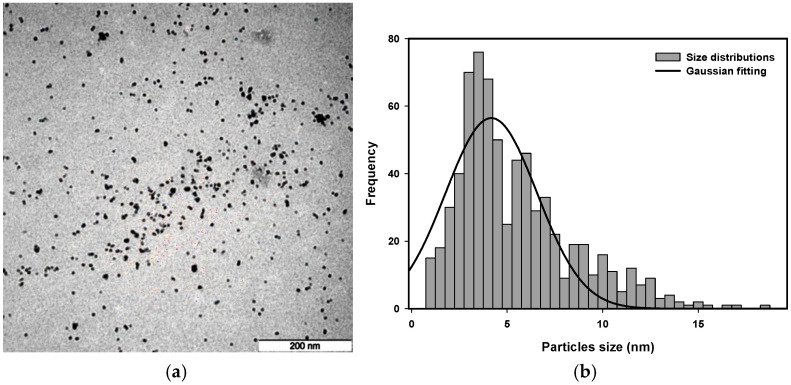
(**a**) TEM micrograph and (**b**) size distribution with Gaussian fitting of Pt nanoparticles of 15.75 × 10^−4^ M irradiated at 90 kGy.

**Figure 13:**
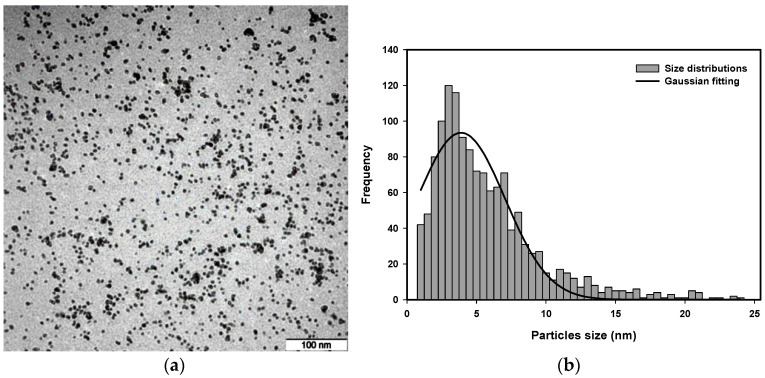
(**a**) TEM micrograph and (**b**) size distribution with Gaussian fitting of Pt nanoparticles of 20.0 × 10^−4^ M irradiated at 110 kGy.

**Figure 14:**
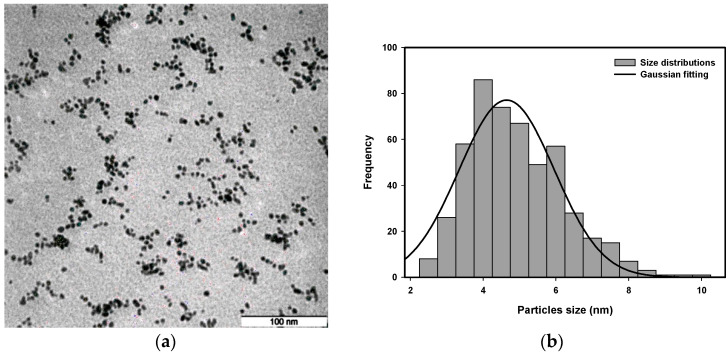
(**a**) TEM micrograph and (**b**) size distribution with Gaussian fitting of Pt nanoparticles of 10.0 × 10^−4^ M irradiated at 80 kGy.

The representative TEM images are presented in Figure 11, Figure 12, Figure 13 and Figure 14 to provide an example of the typical size distributions with Gaussian fitting for concentrations of $10.0\times 10^{-4}\;M$ at a dose of 80 kGy, $11.9\times 10^{-4}\;M$ at a dose of 100 kGy, $15.75\times 10^{-4}\;M$ at a dose of 90 kGy, and $20.0\times 10^{-4}\;M$ at a dose of 110 kGy.

The lower-dose nanoparticles appear to be larger in size than those produced at higher doses. A higher radiation dose will produce a higher nucleation rate, which will result in producing smaller nanoparticles. At the high doses, the nanoparticles exhibit the smallest size and are uniform in shape, with better stabilization provided by PVP. In addition, the effect of precursor concentration on the size of the Pt nanoparticles is clearly evident; higher precursor concentrations can result in larger particle sizes, consistent with an increase in ion recombination probability and a decrease in stabilization efficiency of PVP.

Quantitative analysis of the representative TEM images confirms these trends. For $10.0\times 10^{-4}\;M$ at 80 kGy, the average particle size was determined by Gaussian fitting as 4.66±0.10 nm, with the majority of Pt nanoparticles present in the size distribution range of 4.5–5 nm. For $11.9\times 10^{-4}\;M$ at 100 kGy, the average particle size was 3.56±0.13 nm, with the majority of the particles present in the size distribution range of 3.5–4 nm. For $15.75\times 10^{-4}\;M$ at 90 kGy, the average particle size was 4.17±0.22 nm, with the majority of the Pt nanoparticles present in the size distribution range of 4–4.5 nm. For $20.0\times 10^{-4}\;M$ at 110 kGy, the average particle size was 3.89±0.20 nm, with the majority of the particles present in the size distribution range of 3.5–4 nm. Histograms constructed from the TEM images confirm this trend, with the average size of the Pt nanoparticles decreasing with an increasing radiation dose and the average particle size increasing with an increasing precursor concentration. Therefore, both the radiation dose and the precursor concentration have a substantial influence on the size of the Pt nanoparticles.

Table 1 summarizes representative average particle sizes and conduction band energies for Pt nanoparticles at all precursor concentrations studied. For each concentration, the data correspond to a radiation dose that illustrates the observed trends. These results agree with the UV–Vis absorption analysis and indicate that higher radiation doses yield smaller particles with higher conduction band energies, whereas higher precursor concentrations generally produce larger particles.

These findings are in excellent agreement with the optical absorption and conduction band energy analyses, establishing a strong correlation between synthesis conditions and the electronic and optical behaviors of Pt nanoparticles.

## 4. Conclusions

This study demonstrated a correlation between the process conditions and electronic/optical properties of Pt nanoparticles generated by gamma irradiation of precursors. An increase in the radiation dose is linked to an increase in nucleation sites and formation of smaller and more homogeneous Pt nanoparticles, whereas an increase in the precursor concentration will result in larger particles formed as a consequence of ion recombination. Optical absorption spectra demonstrate a red shift with increased precursor concentrations and blue shifts with higher doses; this can be attributed to the relationship between particle size and electronic transitions. The conduction band energy increases as the particle size decreases, which shows that Pt nanoparticles exhibit quantum confinement effects. In summary, these studies have shown that gamma irradiation allows for the controlled preparation of Pt nanoparticles with specific optical/electronic properties. This research has also furthered our understanding of the structural–property relationships exhibited by Pt nanoparticles produced using gamma irradiation and may enable use in catalytic applications, chemical sensors, or photonic devices.

## Figures and Tables

**Table 1:** Representative particle sizes and conduction band energies of Pt nanoparticles at selected precursor concentrations and radiation doses.

Precursor Conc. (×10^−4^ M)	Dose (kGy)	Avg. Particle Size (nm)	Conduction Band Energy (First Peak) (eV)	Conduction Band Energy (Second Peak) (eV)
5.0	80	4.26	5.82	4.75
10.0	90	3.74	5.79	4.72
11.9	100	3.56	5.80	4.74
15.75	110	3.51	5.80	4.75
20.0	120	3.71	5.80	4.74

## Data Availability

The data presented in this study are available on request from the corresponding author.
